# The CDK7 inhibitor THZ1 alters RNA polymerase dynamics at the 5′ and 3′ ends of genes

**DOI:** 10.1093/nar/gkz127

**Published:** 2019-02-26

**Authors:** Shilpa Sampathi, Pankaj Acharya, Yue Zhao, Jing Wang, Kristy R Stengel, Qi Liu, Michael R Savona, Scott W Hiebert

**Affiliations:** 1 Department of Biochemistry, Vanderbilt University School of Medicine, Nashville, TN 37232, USA; 2 Center for Quantitative Sciences, Vanderbilt University School of Medicine, Nashville, TN 37232, USA; 3 Department of Biomedical Informatics, Vanderbilt University School of Medicine, Nashville, TN 37232, USA; 4 Vanderbilt-Ingram Cancer Center, Vanderbilt University School of Medicine, Nashville, TN 37027; 5 Department of Medicine, Vanderbilt University School of Medicine, Nashville, TN 37232

## Abstract

The t(8;21) is one of the most frequent chromosomal translocations associated with acute myeloid leukemia (AML). We found that t(8;21) AML were extremely sensitive to THZ1, which triggered apoptosis after only 4 h. We used precision nuclear run-on transcription sequencing (PROseq) to define the global effects of THZ1 and other CDK inhibitors on RNA polymerase II dynamics. Inhibition of CDK7 using THZ1 caused wide-spread loss of promoter-proximal paused RNA polymerase. This loss of 5′ pausing was associated with accumulation of polymerases in the body of a large number of genes. However, there were modest effects on genes regulated by ‘super-enhancers’. At the 3′ ends of genes, treatment with THZ1 suppressed RNA polymerase ‘read through’ at the end of the last exon, which resembled a phenotype associated with a mutant RNA polymerase with slower elongation rates. Consistent with this hypothesis, polyA site-sequencing (PolyA-seq) did not detect differences in poly A sites after THZ1 treatment. PROseq analysis after short treatments with THZ1 suggested that these 3′ effects were due to altered CDK7 activity at the 5′ end of long genes, and were likely to be due to slower rates of elongation.

## INTRODUCTION

RNA polymerase is recruited to transcription initiation sites along with several general transcription factors that promote transcription initiation (1,2). Elegant biochemical analyses helped to define the proteins and protein complexes involved and uncovered many mechanistic insights (3). As whole genome approaches are being used in conjunction with small molecules that inhibit specific functions of these factors, a more complete and robust picture is emerging. TFIIH is one such factor and contains ATP-dependent DNA helicase activity to unwind the DNA and allows the initiation of transcription (4,5). In addition, CDK7 forms a trimeric complex with Mat1 and Cyclin H, which is recruited by the ‘core’ TFIIH to phosphorylate the carboxy-terminal domain (CTD) of RNA polymerase II (RNAPII) at Ser5 and Ser7 (6–8). TFIIH works with the negative elongation factor (NELF) and DRB-sensitivity factor (DSIF) to cause the promoter-proximal pause of RNA polymerases (9,10), which coordinates the elongation with 7mG capping of the transcript (8,11–13). CDK9, the enzymatic component of positive transcription elongation factor beta (P-TEFb) mediates the ‘escape’ of RNAPII from the promoter via phosphorylation of the negative elongation factors NELF and DSIF along with the RNAPII CTD at Ser2 (14,15).

The recent development of a very potent and selective covalent inhibitor of CDK7 called THZ1 allowed a more wide-spread analysis of CDK7 in transcriptional control. Initial reports suggested that this compound caused a general loss of RNAPII across a large swath of the genome (16). However, *in vitro* studies using reporter constructs suggested that THZ1 caused RNA polymerase escape from the promoter with a reduction in 7mG capping (17). Nearly 1000 cell lines were tested for sensitivity to THZ1, and T cell leukemia cells that were dependent on the expression of *RUNX1* appeared to be very sensitive. Additionally, THZ1 appeared to selectively target *RUNX1* transcription via a ‘super- enhancer’. Although many acute myeloid leukemia cell lines showed sensitivity, none of these cells contained the t(8;21) translocation, which is dependent on transcription from *RUNX1* regulatory units (16).

The t(8;21) is one of the most frequent chromosomal translocations in acute myeloid leukemia and encodes a fusion protein containing the DNA binding domain of *RUNX1* linked to the majority of the myeloid translocation gene on chromosome 8 (MTG8, also known as eight-twenty-one or ETO; gene name *RUNX1T1*). This chimeric fusion protein binds to RUNX1 DNA elements to control gene expression. Whole genome studies are consistent with earlier findings that MTG8/ETO acts as a transcriptional co-repressor to recruit histone deacetylases with the fusion protein largely repressing RUNX1 target genes (18–23). However, the fusion protein also associates with the co-activator p300 and is acetylated by this histone acetyltransferase (24), and it has also been associated with transcription activation in some cases. In addition, MTG family proteins co-purified in a complex that also contained ‘E proteins’ and CDK9, a component of the positive transcription elongation factor b (P-TEFb). This is likely a large protein complex and the associations between ETO and CDK9 may not be direct and the functional consequence of this association is unclear (25–27).

In this study, we used inhibitors of CDK7 and CDK9 to probe for transcriptional vulnerabilities in t(8;21) AML. We found that two cell lines, as well as patient samples containing the t(8;21), were extremely sensitive to these inhibitors, but not to the CDK4/6 inhibitor palbociclib (PD-0332991). Furthermore, we used PROseq to generate high-resolution maps of active RNA polymerases to assess mechanisms of transcriptional control by CDK7 and CDK9. As expected, inhibitors of CDK9 caused RNA Polymerase II pausing at nearly 80% of the expressed genes in agreement with previous studies demonstrating the importance of P-TEFb in transcriptional elongation (28). Similar effects were observed at enhancers with a general impairment in the production of eRNAs and microRNAs. Conversely, inhibition of CDK7, a critical component of TFIIH caused a loss of paused RNA polymerases at promoters of majority of expressed genes, in agreement with previous studies that used a chemically modified CDK7 mutant at selected reporter genes (9,10). Intriguingly, super-enhancers were only modestly affected after THZ1 treatment within the first hour and the sub-enhancers also showed increased RNA polymerase accumulation. Unexpectedly, THZ1 treatment led to the accumulation of polymerases within the gene body, suggesting slower rates of elongation. Furthermore, CDK7 inhibition altered polymerase dynamics at the 3′ end of select genes, which is also a hallmark of slower RNA polymerase elongation (29,30).

## MATERIALS AND METHODS

### Cell culture

Kasumi-1 cells used in this study were maintained at 37°C with 5% CO_2_. They were cultured in RPMI-1640 (Cellgro) supplemented with 10% heat-inactivated (HI) FetaPlex serum (GEMINI). SKNO-1 cells were cultured in RPMI-1640 supplemented with 10% Stasis FBS (GEMINI) and 10 ng/ml GM-CSF (R&D Systems). RPMI media was supplemented with 2 mM l-glutamine, 100 I.U/ml penicillin, and 100 μg/ml streptomycin. Primary human t(8;21) AML patient cells were cultured with IMDM supplemented with 20% BIT 9500 Serum Substitute (StemCell Technologies), 50 ng/ml SCF (PeproTech), 10 ng/ml IL-6 (PeproTech) and 10 ng/ml IL-3 (PeproTech).

### Drugs used

Flavopiridol (FVP), PHA767491 (PHA), PD-0332991 (Palbociclib, Palbo) and Dinaciclib (Dina) were purchased from Selleck Chemicals. THZ1 was purchased from Apex Bio, MG132 was purchased from Calbiochem and Cycloheximide was purchased from Sigma. All the above drugs were dissolved in DMSO.

### Cell proliferation analysis

Cells were seeded at 2 × 10^5^ cells/ml on the day of the experiment, and treated with increasing doses of the drugs (FVP, Dina, PHA, Palbo and THZ1) for three consecutive days. Human primary AML cells were seeded at 5 × 10^5^ cells/ml in a 96-well plate. Cell proliferation rate was measured on each day using alamar Blue assay (cat # DAL1100, Life Technologies) according to the manufacturer's instruction. Briefly, 100 ul of cell culture was transferred to a 96-well plate, 10 ul of alamar Blue was added. Plates were incubated at 37°C for 4 h and read at 590 nm emission wavelength. The plates were analyzed on a Biotek plate reader. At the same time, viable cells were quantified by Trypan Blue dye exclusion assay.

### Assessment of apoptosis

Cells were seeded at 2 × 10^5^ cells/ml and treated with either DMSO or 400 nM FVP, 5 uM PHA, 5 uM Palbo, 25 nM Dina and 400 nM THZ1 for 6 h and apoptosis was analyzed using a FITC-AnnexinV/PI Apoptosis Detection kit (cat # 556547, BD Pharmingen). Flow cytometry data were analyzed using the FlowJo software.

### Co-Immunoprecipitation

Kasumi-1 cells were washed with cold PBS, lysed with NETN buffer (100 mM NaCl, 20 mM Tris pH 8.0, 0.5 mM EDTA, 0.5% NP-40), and pulse sonicated before centrifugation. Lysates were incubated with CDK9 antibody (Santa Cruz) or normal IgG (Santa Cruz) in the cold room for overnight. 10% of lysate was saved as input. On the next day, protein complexes were pelleted using protein G magnetic beads (Millipore) and subjected to SDS-PAGE and western blot.

### Western blot

To analyze protein expression, cell pellets were lysed with RIPA buffer (50 mM Tris pH 8.0, 150 mM NaCl, 1% NP-40, 0.5% sodium deoxycholate, 0.1% SDS) and sonicated briefly before centrifugation. Equal amount of protein was subjected to SDS-PAGE and western blot. Membranes were incubated with primary antibodies against MYC (clone 9E10), RUNX1-ETO, GAPDH, (Santa Cruz), Tubulin (Abcam). Signals were amplified with IRDye secondary antibodies (LI-COR Biosciences) and detected using Odyssey imager.

### ChIP and quantitative RT-PCR

ChIP and qRT-PCR were performed as described in (31). Briefly, Kasumi-1 cells were seeded at 5 × 10^5^ cell/ml on the day of the experiment and treated with DMSO or 400 nM THZ1 for 1hr. Cells were then crosslinked with 1% formaldehyde at room temperature for 10 min with gentle rocking. Crosslinking was quenched by 125 mM glycine. Cells were then washed with cold PBS twice before lysing with 1% SDS lysis buffer and sonicated with Bioruptor. Chromatin was diluted 1:10 and incubated with anti-phospho Ser2-RNAPII (ab5095) or Normal Rabbit IgG (sc-2027) and Dynabeads protein A/G magnetic beads overnight at 4°C. Immunoprecipitated complexes were washed, reverse-crosslinked and proceeded for DNA isolation. 2 ul of ChIP DNA was used for quantitative RT-PCR. Data were calculated relative to input and IgG for normalization and background reduction.

### Nuclei isolation

Kasumi-1 cells were treated with CDK inhibitors and collected at desired time points. 25 million cells were washed with cold PBS, resuspended in 10 ml of cold swelling buffer (10 mM Tris pH7.5, 2 mM MgCl_2_, 3 mM CaCl_2_, 300 mM Sucrose, protease inhibitors), and left on ice for 5 min. Cells were pelleted by centrifugation and resuspended in 2 ml lysis buffer (swelling buffer + 10% glycerol + 0.1% Triton X-100, and protease inhibitors) and left on ice for 5 min followed by dounce homogenization for 50 times. Another 5 ml of lysis buffer was added and nuclei were pelleted by centrifugation. Nuclei were sequentially washed with 5 ml of lysis buffer and 1 ml of freezing buffer (50 mM Tris–Cl pH 8.3, 40% glycerol, 5 mM MgCl_2_, 0.1 mM EDTA). Pelleted nuclei were re-suspended in freezing buffer at the density of 2 × 10^7^/100 ul, and stored at −80°C.

### PROSeq library preparation and data analysis

Nuclear run-on assays for PROseq and the library preparation was performed as described in (32) PROseq libraries were submitted to Vanderbilt Technologies for Advanced Genomics (VANTAGE) for sequencing on Illumina HiSeq2000. Data analysis for PROseq was done as described in (31), Briefly after adapter trimming and low-quality sequence removal by cutadapt, PROseq reads longer than 15bp were reverse-complemented using FastX tools. Reverse complements of the trimmed reads were aligned to the human genome hg19 using Bowtie2. Reads mapped to rRNA loci and reads with mapping quality <10 were removed. Bam files were given to NRSA (33) to estimate RNA polymerase abundance in promoter-proximal and gene body regions of genes, to calculate pausing index and pausing index alterations, and to detect enhancers and quantify eRNA changes. HOMER was used to draw the meta-analysis plots (34).

### Statistical analyses

DESeq2 was used to compare promoter-proximal and gene-body regions between groups, while Cochran–Mantel–Haenszel's (CMH) test was used to compare pausing index between groups (33). ANOVA test was used for Annexin V quantitation. *P* values <0.05 were considered as statistically significant. For Apoptosis, results were presented as mean ± SEM.

### RNASeq and analysis

PolyA+ RNA was enriched for the library preparation from the corresponding treated samples that were also used for PROseq experiment. RNA was submitted to VANTAGE for sequencing. RNAseq analysis was done as described in (35).

### PolyA seq

Total RNA was isolated after 1hr THZ1 treatment from Kasumi-1 cells. PolyA-seq libraries were prepared as described in (36) with several modifications. DNA libraries of ∼500 bp were sequenced by PE sequencing on Illumina HiSeq3000. Sequencing files were preprocessed and mapped to hg19 genome and the identification, quantification and analysis of differential poly A site selection/usage were performed as described in (37).

### Gene set enrichment analysis

We acknowledge our use of the gene set enrichment analysis, GSEA software, and Molecular Signature Database (MSigDB) (http://www.broad.mit.edu/gsea/) (38).

## RESULTS

### Cell lines containing the t(8;21) are sensitive to CDK7 or CDK9 inhibition

**Figure 1.**
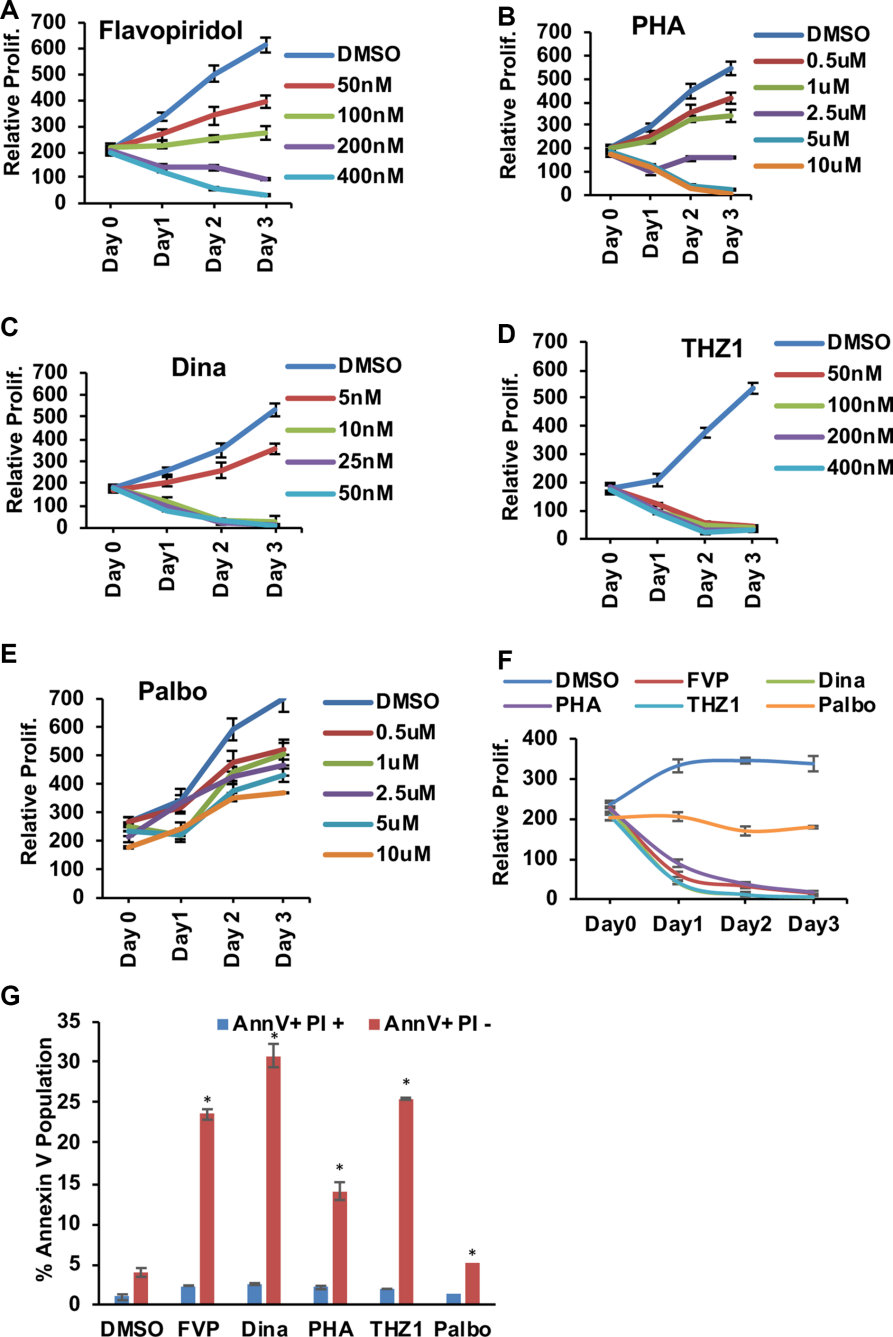
AML containing the t(8:21) are extremely sensitive to CDK inhibitors. (**A–C**) Alamar blue assays were used to assess the cell proliferation of SKNO-1 cells to evaluate the dose response of CDK9 inhibitors flavopiridol (FVP), dinaciclib (Dina) and PHA767491 (PHA) were used in increasing concentrations. (**D**) Alamar blue assay for the CDK7 inhibitor THZ1 and (**E**) CDK4/6 inhibitor palbociclib (Palbo). (**F**) Combined growth curve for Kasumi-1 cells from Alamar blue assays. The graph represents the drugs used at the concentrations used in the final PROseq experiment (FVP (400 nM), PHA (5 uM) Dina (25 nM), THZ1 (400 nM) and Palbo (5 uM). For Alamar blue assay each experiment was performed at least three times (*n* = 3). Data are plotted as mean ± SEM. Error bars represent standard error of means (SEM) from three experiments. (**G**) Quantitation of Annexin V/PI staining for all the drugs used at the same concentration as in C and incubated for 6 hrs. Data are plotted as mean ± SEM. Error bars represent standard error of means (SEM). Asterisk (*) indicate *P*-values <0.05.

AML containing the t(8;21) translocation show exceptional sensitivity to flavopiridol and dinaciclib, two broad-spectrum cyclin-dependent kinase (CDK) inhibitors that have high potency against CDK9, both *in vitro* and in clinical trials (39,40). Proteomic studies suggested that MTG/ETO family members not only regulate transcription via the recruitment of HDACs, but also associate with a complex that includes CDK9 (27,41–43). This association of AML1-ETO (AE) and CDK9 suggested that cells containing the t(8;21) might be sensitive to inhibitors that affect the decision point after RNA polymerase transcriptional initiation, where the polymerase pauses and/or elongates to complete the mRNA. In addition, a recently described CDK7 inhibitor, THZ1, suppressed *RUNX1* expression in T–ALL cells (16), and additionally, mutations in Cyclin D2 suggested that t(8;21) might up-regulate CDK4/6 activity to circumvent the inhibition of cell cycle progression by AML1-ETO (AE) expression (44–47). Therefore, we tested the sensitivity of two t(8;21)-containing AML cell lines, SKNO-1 (Figure 1A–E) and Kasumi-1 (Figure 1F), to flavopiridol (FVP, broad spectrum CDK inhibitor), dinaciclib (Dina, a more potent broad spectrum CDK inhibitor), PHA767491 (PHA, a more selective CDK9 inhibitor), THZ1 (CDK7 inhibitor) and palbociclib (palbo, CDK4/6 inhibitor). Both of these t(8;21) cell lines showed dramatic sensitivity to dinaciclib and flavopiridol in viability assays, with dinaciclib showing low nanomolar potency. PHA767491, a more selective CDK9 inhibitor, did not inhibit growth until the low micromolar range (5μM) where it could inhibit other CDKs, suggesting that CDK9 inhibition alone was not sufficient to kill these cells.

Both Kasumi-1 and SKNO-1 cells were very sensitive to THZ1 in the nanomolar range (Figure 1D and F). Although CDK7 is also the cyclin activating kinase (CAK) for CDK4/6, even at ten-fold higher levels the CDK4/6 inhibitor palbociclib had only modest effects on cell growth (Figure 1E). This was unexpected as palbociclib had been suggested as a potential therapeutic in t(8;21) AML (44). By contrast, all of the ‘transcriptional CDK’ inhibitors triggered a far more rapid and deep apoptotic response as measured by flow cytometry to detect annexin V, whereas palbociclib had almost no effect on cell death (Figure 1G). AML patient samples containing t(8;21) were also extremely sensitive to these inhibitors (Supplementary Figure S1A–E). We also tested leukemia cell lines that did not contain t(8;21) translocation and these cells showed variable sensitivity to THZ1 (Supplementary Figure S1F–I).

**Figure 2.**
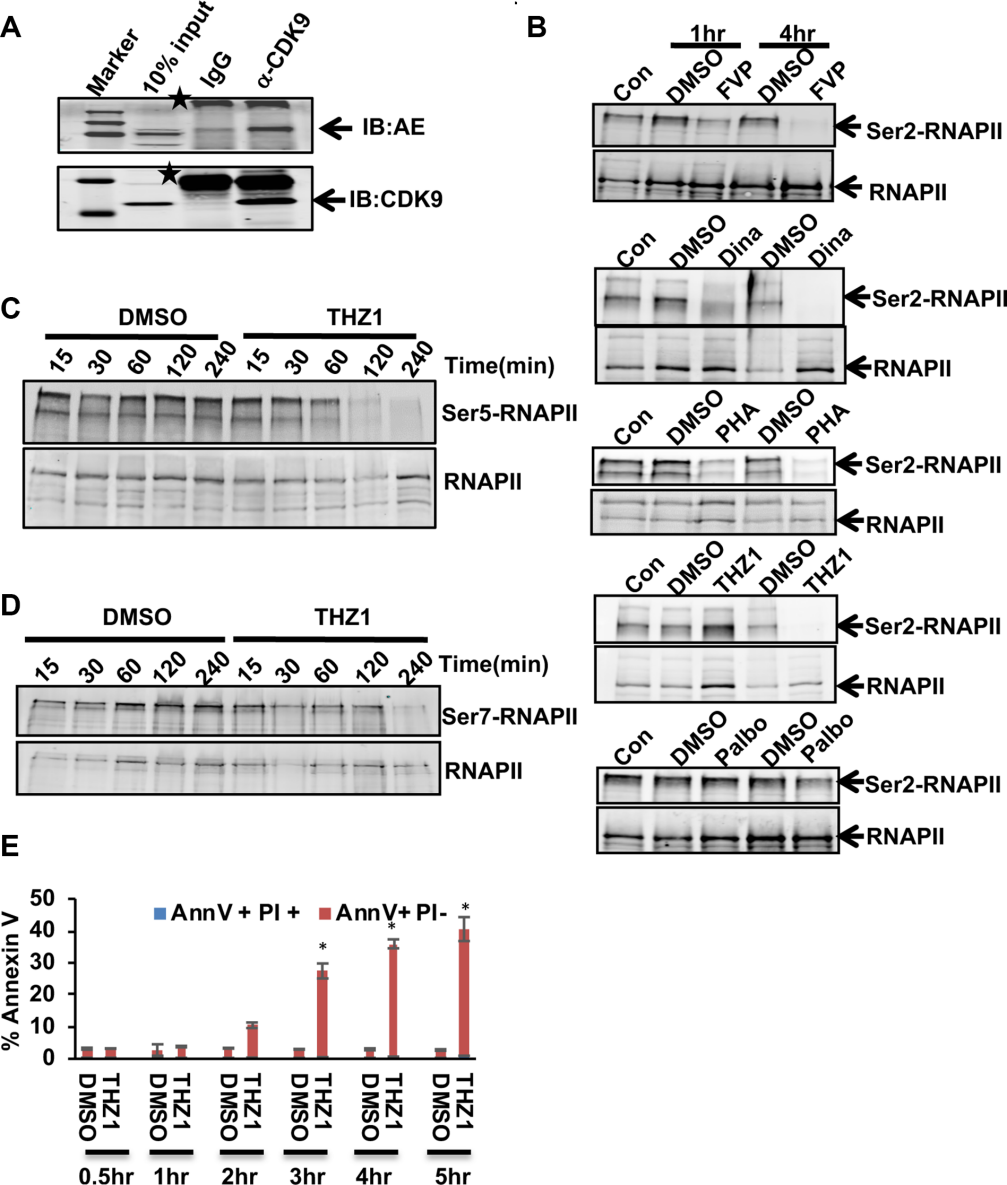
CDKi inhibits RNA Polymerase CTD Phosphorylation. (**A**) Co-IP showing AML-ETO (AE) fusion protein from Kasumi-1 cells extracts was precipitated using anti-CDK9. Asterix (*) depicts non-specific bands. (**B**) CDKi treated Kasumi-1 cell extracts were tested for phospho-Ser2 RNA polymerase II levels by western blot. (**C, D**) THZ1 treated Kasumi-1 cell extracts from the indicated time-course experiment were tested for Ser5 and Ser7 phosphorylated RNA polymerase II levels by western blot. (**E**) Quantitation of Annexin V/PI staining analysis of Kasumi-1 cells treated with THZ1 at 400 nM for 30, 60 120 and 240 min. Asterisk (*) indicate *P*-values <0.05

Next, we used co-immunoprecipitation assays to confirm that a small amount of endogenous AML1-ETO (AE) associates with CDK9 in Kasumi-1 cellular extracts (Figure 2A). We also confirmed the action of these kinase inhibitors by examining RNAPII CTD phosphorylation levels. As expected, the CDK9 inhibitors caused a marked decrease in the phosphorylation of the Ser-2 form of RNAPII CTD after just 1–4 h of treatment whereas palbociclib, a CDK4/6 inhibitor, had no effect (Figure 2B) (48–50). THZ1 also impaired phosphorylation of Ser2 of the RNAPII CTD, but the effects were delayed relative to the inhibition of Ser5 phosphorylation (Figure 2C), which is consistent with the role of CDK7 as the CDK activating kinase (CAK) that activates both cell cycle kinases (CDK4/6) and CDK9 (9,16). THZ1 impaired Ser7 phosphorylation by 4 h of treatment emphasizing that Ser5 is preferred by CDK7 (Figure 2D). Remarkably, CDK7 inhibition triggered the appearance of annexin V positive cells within 2 h, and by 4 h nearly half of the cells were annexin V positive (Figure 2E).

*RUNX1* was reported to be a key gene whose expression was inhibited by THZ1 (16). However, THZ1 had little impact on the level of expression of the fusion protein driven by the RUNX1 promoter/enhancer elements in the first 4 h of treatment, which is prior to widespread apoptosis (Supplementary Figure S2A). This result may suggest differences in *RUNX1* control elements in T cell acute lymphocytic leukemia and AML, but indicates a novel mechanism of action for this compound in AML. *MYC* has also been suggested to be a key target of THZ1, but we noted a modest increase in *MYC* mRNA levels and the levels of MYC protein were largely unaffected in the first 2 h but declined 4 h after addition of THZ1 (Supplementary Figure S2B and C). Given that CDK7 regulates the CDKs that control MYC stability, we used the proteasome inhibitor MG132 in combination with cycloheximide (CHX) to inhibit new protein synthesis to assess MYC protein stability. MYC levels were not appreciably destabilized even at 4 h post treatment with THZ1, suggesting that although CDK7 regulates RNAPII pausing, the effects on MYC were indirect and required longer exposure to the drug (Supplementary Figure S2B and C).

### CDK7 inhibition alters promoter-proximal pausing

While TFIIH is typically associated with RNA polymerase initiation and pausing (9,51), THZ1 was credited with causing the loss of RNA polymerase genome-wide as assessed by ChIP-seq (16). In contrast, THZ1 caused the loss of pausing in *in vitro* assays on reporter constructs, and it also impaired the addition of the 7mG cap and impaired productive elongation (10,17). We used precision global run-on transcription coupled with deep sequencing (PROseq) (32,52) to assess genome-wide effects on RNA polymerase pausing dynamics to dissect the mechanisms underlying these observed phenotypes. Kasumi-1 cells were used, as these cells are similar to patient samples in transcription factor occupancy and histone modification profiles (21,22), making this cell line an excellent model to study the effects of these compounds. To exclude any secondary or compensatory effects, we assessed the early events of transcription following a 1 h treatment with all the CDK inhibitors used in this study.

A bioinformatics pipeline called Nascent RNA Sequencing Analysis (http://bioinfo.vanderbilt.edu/NRSA, (33) was used to analyze the PROseq data over a 50 bp sliding bin ±500 bp of the transcription start site (TSS) to assess the ratio of the read densities in the promoter-proximal region of the transcription start site (TSS) versus the ‘gene body’ (gb) to define a ‘pausing index’ (Supplementary Figure S3A) (52–54). While this pausing index was calculated within a dataset and is not affected by normalization, for the calculation of gain or loss of gene body polymerase accumulation, the PROseq data were either normalized using exogenously added biotinylated RNAs (i.e. ‘spike-in controls’), or the 3′ ends of genes longer than 600kb where the polymerase would not be affected within the time course (i.e. polymerase travels 2–4 kb/min, assessed in our earlier study) (28,31).

**Figure 3.**
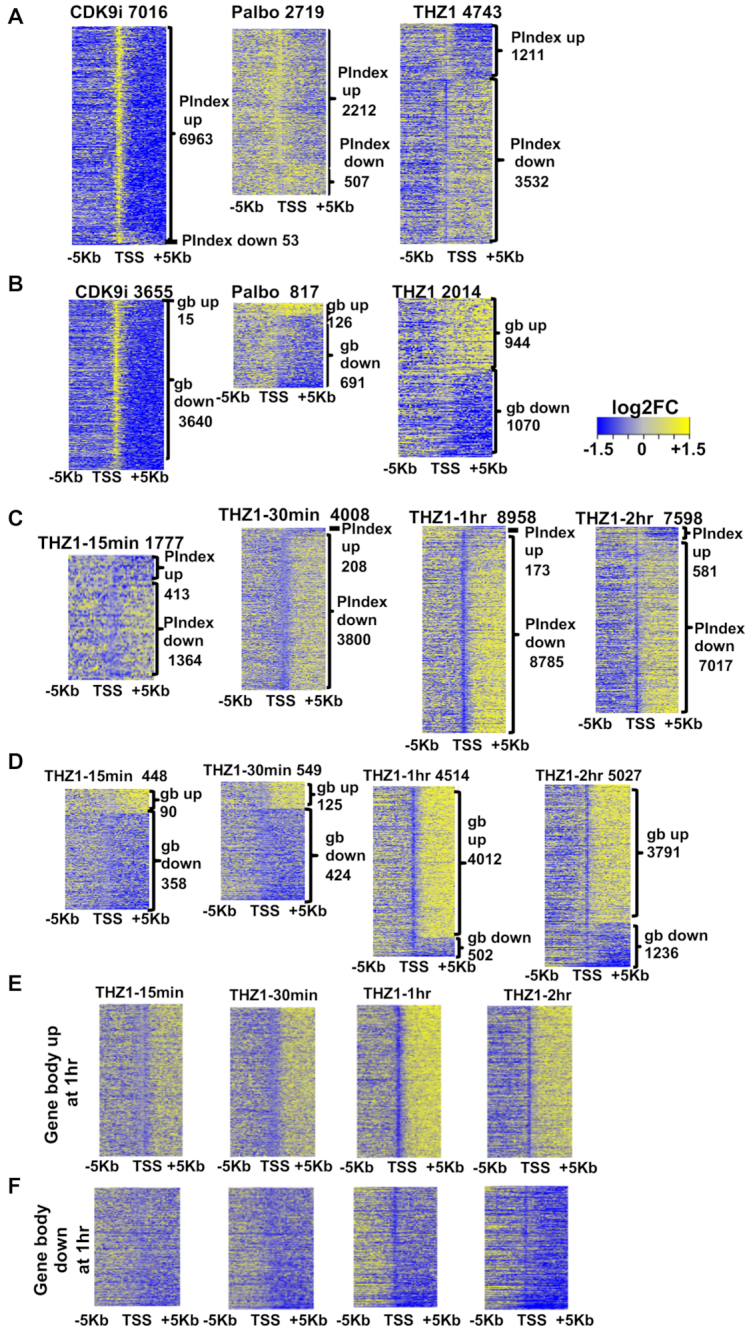
CDKi affect promoter-proximal RNA polymerase pausing. (**A**) log_2_ transformed fold change (log_2_FC) values for the affected genes are ranked according to the pausing indices (log_2_FC). Then log2FC PROseq read counts in 200 bp bins ± 5 kb around the TSS for these genes from each indicated drug were depicted as heatmaps. The labels on the top indicated the drug used and the number of refseq genes in the heatmap. CDK9i represent the common genes affected by all the three CDK9 inhibitors (FVP, Dina and PHA). The labels on the side indicate the number of genes that gained or lost pausing of RNA polymerase II at promotor-proximal regions (P.Index). (**B**) Similar strategy as in (A) was employed in drawing the heatmaps but the genes were ranked according to the (log_2_FC) of gene body read counts from PROseq. The numbers on the side represent the number of the refseq genes that gained or lost polymerase in the gene body. (**C**) Time course of THZ1 treatment. log2FC of PROseq read counts in 200 bp bins ± 5 kb of the TSSs for the THZ1 affected genes from each indicated time-point of the time course experiment (15 min, 30 min, 1 h and 2 h with THZ1 treatment at 400 nM). Genes are ranked based log_2_ transformed fold change values (log_2_FC) of pausing index. (**D**) heatmaps of genes ranked according to the log_2_FC of gene body read counts using similar strategy as in A. the log_2_FC of read counts in 200 bp bins ± 5 kb of the TSS for the genes that either increased gene body polymerase density (**E**) or decreased gene body polymerase density (**F**) at 1 h post treatment. These same genes were then plotted from each of the indicated time-point as heatmaps ± 5 kb from the TSS (up-regulated 4012 genes; down-regulated 502 genes, from **D**).

As expected from previous studies of flavopiridol, each of the CDK9 inhibitors caused a dramatic increase in the pausing index of over ∼6000 genes with a loss of elongation, whereas only about 50 genes consistently showed a decrease in pausing index (Figure 3A, first panel and S3B). Taken together, these observations emphasize the role of CDK9 as a positive regulator of transcriptional elongation and its inhibition had dramatic effects on the majority of expressed genes in the first 1 h of treatment including *MYC* and *BCL2*, which likely contribute to cell death of Kasumi-1 cells. While the pausing ratio does not depend on normalization (it is a ratio within a single gene), it emphasizes the promoter-proximal effects. Therefore, we further compared normalized RNA polymerase densities in the gene body (gb) for assessing the density of active polymerases in these genes. All three inhibitors of CDK9, yielded dramatic reductions in gene body transcription on roughly 40% of the expressed genes, as the polymerases accumulated within the first 100–200 bp of the gene (i.e. blocked elongation) (Figure 3B first panel).In contrast to the effects of CDK9i, THZ1 treatment generally yielded the opposite effect in which there was a loss of promoter-proximal pausing at 3532 genes and an increase/gain in polymerase pausing at 1211 genes as assessed by pausing ratio after only 1 h of treatment that was largely due to loss of polymerase in the gene body rather than an increase at the start site (Figure 3A, third panel). These results are consistent with previous mechanistic analyses of the effects of THZ1 on reporter plasmids and were foreshadowed by the analysis of the function of TFIIH in establishing promoter proximal pausing of RNA polymerase (9–10,12,17). When transcription through the gene body was assessed, THZ1 treatment increased the polymerase density of 944 genes, whereas 1070 genes showed a decrease in polymerase density within the gene body (Figure 3B, third panel). The general trend upon THZ1 treatment was a loss of RNA polymerase pausing at the promoter with increased gene body polymerase (Figure 3A and B, third panels).

*MYC* is a gene that is regulated by pausing and that controls cell metabolism (55) and is targeted by THZ1 in some cells types, along with *MYCN* (56,57). However, THZ1 treatment had little effect on either *MYC* or *MYCN* loci with a modest increase of RNA polymerases within the gene body, but a distinct reduction in the polymerase occupancy at the promoter proximal site for *MYCN* was observed (Supplementary Figure S3C, dotted box). Likewise, the *RUNX1* promoter showed an increase in RNA polymerase rather than a decrease with THZ1 treatment (Supplementary Figure S3C). The above findings were in stark contrast to CDK9i treatment with PHA where there was a modest effect in the levels of RNA polymerase without a change in promoter-proximal pausing at the *MYC* promoter (Supplementary Figure S3D. At *MYCN*, the polymerase accumulation around the promoter site was accompanied by decreases in the gene body (Supplementary Figure S3D).

Because CDK7 is also the cyclin activating kinase (CAK) that is needed for activation of CDK4/6 and D type cyclins have been reported to regulate transcription (58,59), we also tested the CDK4/6 selective inhibitor palbociclib (Palbo) using PROseq. Surprisingly, inhibition of these G_1_ phase CDKs had modest effects on transcription with only about 500 genes showing a reduction in polymerases at promoter-proximal sites (Figure 3A, Palbo, second panel), while 2212 genes showed an increase in promoter-proximal pausing (Figure 3A, Palbo). After normalization and using gene body transcription rates for analysis, 691 genes showed a reduction in transcription rate (Figure 3B, Palbo). Even within this set of genes the effects were relatively modest suggesting that the majority of these effects were due to a general metabolic pathway perturbation. This was likely due to reduced phosphorylation of the retinoblastoma tumor suppressor (Rb) causing suppression of E2F transcriptional targets (Supplementary Figure S3E). Therefore, it appears that the loss of promoter-proximal pausing observed with THZ1 is due to inhibition of its direct target CDK7 and not indirect effects on CDK4/6 (60,61).

Given the rapid effects of THZ1 on polymerase dynamics at 1 h, we repeated the PROseq analysis at 0, 15 min, 30 min, 1 h and 2 h to determine the mechanism by which CDK7 controls polymerase dynamics. By plotting the changes in polymerase occupancy using either pausing index (Figure 3C) or normalized changes in the gene body (Figure 3D), we observed changes in promoter-proximal polymerase pausing as early as 15 min with an expanding number of genes showing a loss of promoter-proximal pausing at 30 min and 1 h, confirming the effects observed previously (Figure 3A and B). It is notable that there were small differences in potency in batches of THZ1 such that the effects observed at 1 h with some batches were not as dramatic as with other batches (e.g. note that the number of genes affected in Figure 3A versus C). These small differences in potency and timing did not affect the overall conclusions that most of the expressed genes showed a statistically significant change in pausing index within the first 1–2 h of THZ1 treatment (Figure 3C).

When focusing on the changes within the body of genes, we noted that about half of the genes showing pausing index changes met the 1.5-fold cut off applied to changes in gene body occupancy (Figure 3B and D). At 15 min post-treatment there were few genes showing a significant increase in polymerase density with more genes showing lower amounts of polymerase within the gene body. However, over time the number of genes with increased polymerase density in the gene body continued to grow to 4012 at 1 h (Figure 3D). By graphing all of the genes showing accumulation of polymerase in the body of genes that went up or down at 1 h post-treatment with THZ1 and examining the polymerase occupancy at 15 and 30 min, we noted that for genes with increased polymerase density after THZ1 treatment there was an area around the transcriptional start site that was devoid of polymerases (e.g. at 30 min) and that this area narrowed by 1 h and further narrowed at 2 h (Figure 3E). This may represent engaged polymerases being rapidly released from the promoter-proximal pause (15 and 30 min) and re-initiation without pausing at these promoters at later times (1–2 h) to re-establish a higher steady state level of polymerase occupancy. Moreover, the narrowing of the ‘gap’ in polymerases around the start site is consistent with the possibility that THZ1 caused a slowing of polymerase elongation, yet the polymerases still reached the 3′ ends of genes (see Figure 7).

Because RNAPII moves at about 2–4 kb/min (31), these data implied that THZ1 did not affect the rate of transcription initiation of most genes, because continued initiation was required to repopulate the first 5 kb of these genes (the area around the TSS in Figure 3C and D). In contrast, for those genes that were downregulated at 1 h there was a general loss of polymerases over time both at the promoter-proximal site and in the gene body. This could represent a subset of genes that failed to initiate or reinitiate transcription in response to THZ1 or in which the polymerase failed to engage in active elongation (Figure 3F).

A meta-analysis of polymerases around the start sites for the genes with increased polymerase density at 1 h (using the 1 h gene set from Figure 3DSupplementary Figure S3F), showed a progressive loss of pausing over time. Motif analysis did not identify TATA-box selectivity within the promoter of genes affected by THZ1 (Supplementary Figure S3G), but ETS family motifs were among the most frequently found, which is consistent with a small consensus binding site that is found on most genes (Supplemental Table S1). Analysis of Kasumi-1 ChIPseq data for H3K4me3, H3K27Ac, H4K12Ac and H5K5Ac marks indicated that higher levels of these marks were associated with the genes affected by THZ1, which implies that a more open local chromatin configuration and higher level of gene expression correlated with the loss of pausing (Supplementary Figure S3H–K). Therefore, we did not identify any gene-specific selectivity for THZ1 treatment, which is consistent with the role of TFIIH as a general transcriptional regulator.

**Figure 4.**
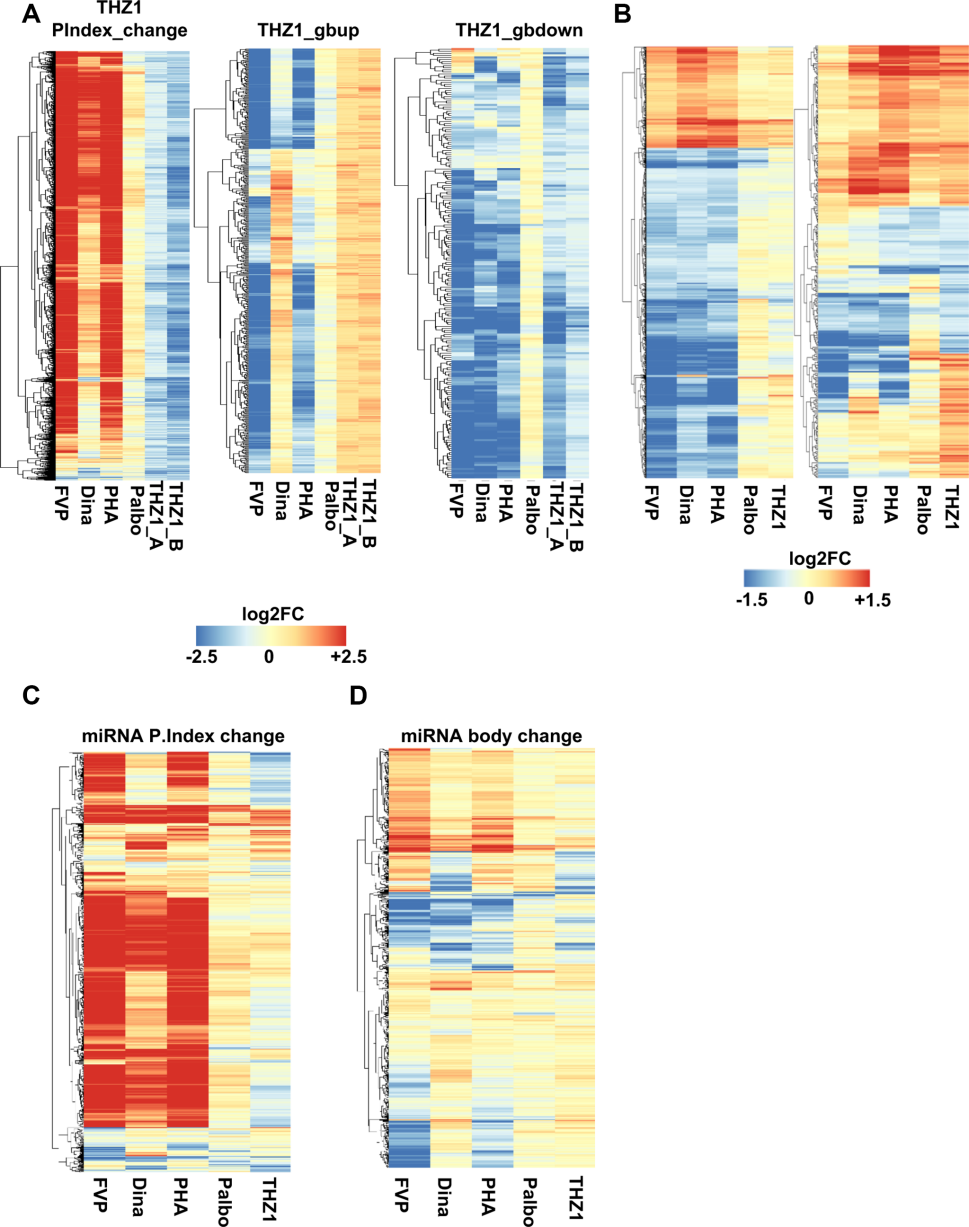
Unsupervised clustering analysis on genes affected by CDK Inhibitors. (**A**) Unsupervised clustering analysis on commonly overlapped genes that lost promoter proximal pausing after 1 h THZ1 treatment (Figure 3A) and 1 h time-point from the THZ1 time course experiment (Figure 3C) in comparison to the other CDK inhibitor treatments based on the pausing index change (PIndex change, left panel). Similar analysis was performed on genes that either increased polymerase density in the gene body or vice versa after THZ1 treatment. (**B**) Left panel: unsupervised clustering analysis of the commonly overlapped, differentially expressed genes from the RNAseq data for 1 h CDK9i (FVP, Dina and PHA) treated samples as compared to palbociclib or THZ1 treated samples processed at the same time as the PROseq samples. Right panel: unsupervised clustering analysis of the differentially expressed genes from the RNAseq data for 1 h THZ1 treated samples. The expression of these genes is then shown after a 1 h treatment with the other inhibitors. (**C**) log_2_ transformed fold change values of pri-miRNA expression relative to DMSO are shown as heatmaps for the pri-miRNAs affected by the CDKi ranked according to the pausing indices (C) and pri-miRNA gene body (**D**).

To gain insights into mechanisms between CDK9i and CDK7i, we employed unsupervised hierarchical clustering on commonly overlapped genes from the 1hr time points in the THZ1 PROseq experiments (See Figure 3A and C, third panels) and compared them to each of the individual CDK treatments based on log2 transformed values of their respective pausing index change (Figure 4A, left panel) and gene body density changes (Figure 4A. middle and right panel). This analysis showed that with CDK9i a strong clustering of genes that had increase in promoter-proximal pausing (red) and concomitant decrease in gene body (blue), whereas THZ1 had the inverse effect (light blue, Figure 4A) and palbociclib had modest effects (Figure 4A). We also performed RNAseq experiments using these compounds to compare the PROseq data to the steady state pools of mRNA. Similar clustering analysis on RNAseq data using the genes that were affected by CDK9i (Figure 4B, left panel) showed that the block in elongation led to reduced levels of mRNA for a large number of genes (blue). Those mRNAs that were affected by THZ1 (Figure 4B, right panel) showed that in the first hour of treatment there was a group of genes that were induced with all of the compounds, suggesting a stress response.

Because PROseq yields the entire transcribed genome, we were also able to examine microRNAs in our study (62–64). Examination of the 721 pri-miRNAs expressed in Kasumi-1 cells (528 Intragenic and 193 Intergenic) showed that THZ1 treatment caused a loss of pausing at 185 pri-miRNAs and 83 showed accumulation of promoter-proximal polymerases in the first hour of treatment. Unsupervised hierarchical clustering analysis for the drug treatments using pausing index and pri-miRNA body densities (Figure 4C and D) showed that the CDK9i largely suppressed miRNA expression (red, increase in pause), whereas CDK7i had the opposite effect. Interestingly, miRNA MIR155, which has a putative role in leukemogenesis and that is often up-regulated in B-cell malignancies, showed an accumulation of polymerase after THZ1 treatment but was repressed by CDK9i (65,66) (Supplementary Figure S4).

### CDK7 inhibition modestly affects super-enhancers

**Figure 5.**
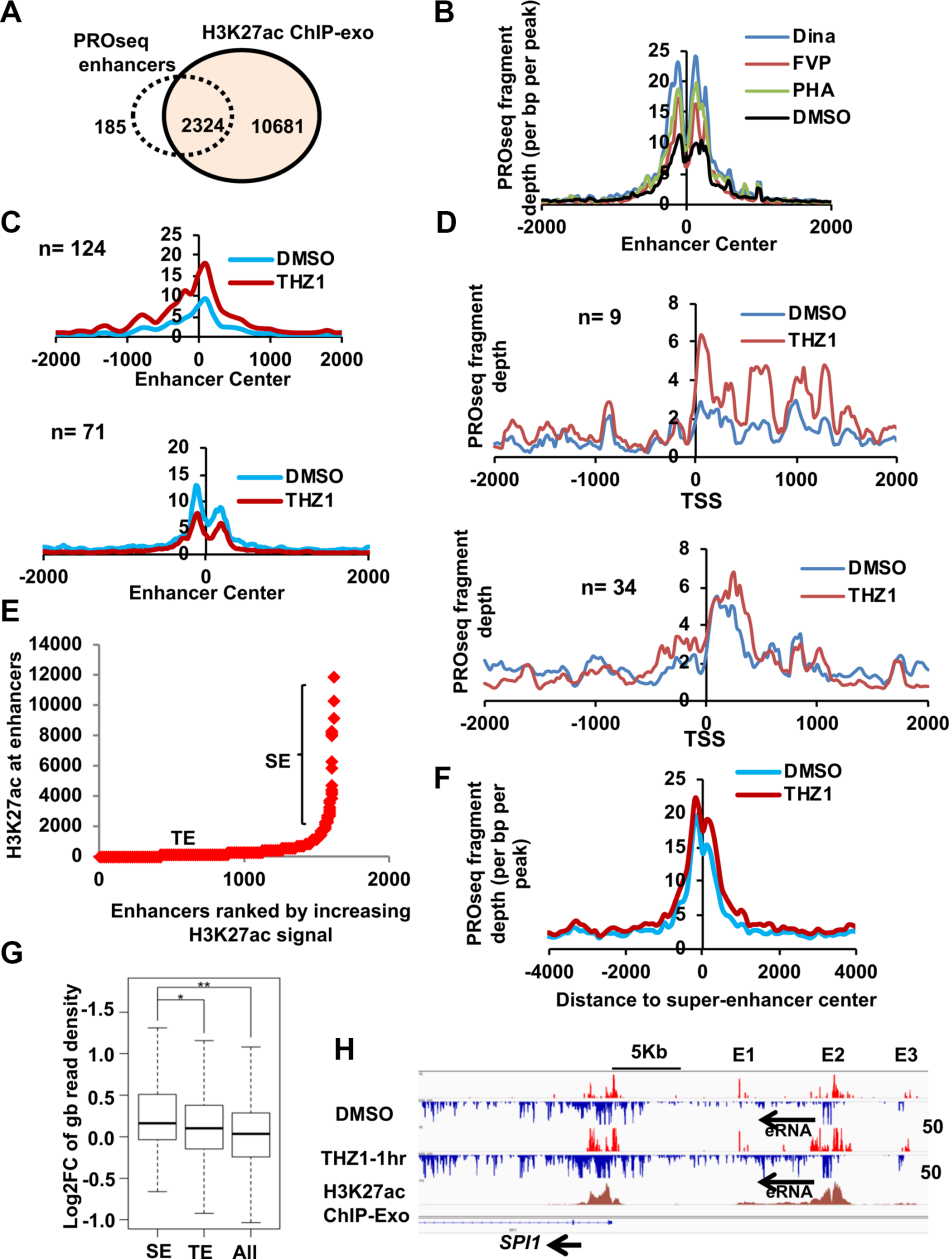
CDKi affects the transcription of eRNA from Enhancers and Super-enhancers. (**A**) Venn diagram showing the overlap between PRO-seq called enhancers and H3K27ac ChIP-exo peaks from Kasumi-1 cells indicates that the majority of the PRO-seq called enhancers were marked by H3K27ac. See also Supplemental Figure S5A (B, C) Meta-analysis plots for the PROseq signal around enhancer centers are shown for commonly overlapped enhancers from CDK9i (**B**). (**C**) Similar analysis as in (B) but using 124 enhancers with increased polymerase around the enhancer center (top panel) after THZ1 treatment or 71 enhancers with decreased polymerase around enhancer center (bottom panel). See also Supplemental Figure S5B for the genes associated with these enhancers. (**D**) Meta-analysis of long eRNA from THZ1 treatments that had either an increase (top panel) or a decrease in gene body (bottom panel) showed a concomitant increase in polymerase density around the start site of the long eRNA. See also Supplemental Figure S5C and S5D for the general distribution of these long eRNA. (**E**) Hockey stick plot of PROseq identified intergenic enhancers ranked according to the increasing amounts of H3K27ac signal. Those enhancers that had high H3K27ac signal and above the slope of 1 were termed ‘super-enhancers’ (SE) and the ones below the slope of 1 were considered typical enhancers (TE). (**F**) Metaplot of PROseq signal at the super enhancer regions after THZ1 treatment showed a slight accumulation of RNA polymerases at the enhancer center in comparison to DMSO. Metaplots were generated using HOMER. (**G**) Box plots of nearest neighbor genes associated with SE and TE against all genes (All) were plotted for the log_2_FC of the normalized PROseq read counts in the gene body. *P = 0.02, **P = 6.96e–05. Comparison testing was done by Kolmogorov–Smirnov (KS) test. (**H**) IGV browser screenshots of genome tracks for a ‘super-enhancer’ located downstream of *SPI1* that was affected by THZ1 treatment.

PROseq also yields information on enhancer RNAs, long non-coding RNAs and transcription from the start sites of enhancers. In fact, ENCODE and nascent RNAseq data show that active enhancers produce 5′ capped transcripts, have the structural architecture of promoters, and have defined transcriptional start sites that produce bi-directional transcription (52,67–71). Therefore, we examined active enhancers and ‘super-enhancers’ given that the RUNX1 super-enhancer was pinpointed as a key target for THZ1 in T cell acute leukemia (16). By overlapping our PROseq datasets with H3K27ac ChIP-exo data (31), we identified over 2300 intergenic enhancers in Kasumi-1 cells and these enhancers were mostly marked by H3K27ac and H3K9ac (21) (Figure 5A, and Supplementary Figure S5A). By using a 1.5-fold cut-off change between CDKi treated sample and the control for the PROseq read densities at the enhancer center (typically the start site for bi-directional transcription), we found that inhibition of CDK9 for 1 h caused polymerase accumulation at about ∼1000 enhancers, which is likely a sign of impaired elongation (signal peak around the start site) (Figure 5B). In the case of THZ1 treatment although only 194 out of the 2000 intergenic enhancers were changed after 1 h of treatment, and 124 of these enhancers reached the 1.5-fold change cut off and had increased polymerase signal around the enhancer start site (Figure 5C, top panel). 71 enhancers had decreased polymerase signal at the start site (Figure 5C, bottom panel. The genes associated with the 124 enhancers had increased gene body density (Supplementary Figure S5B, left panel) and for the 71 enhancers the associated genes showed no changes in the gene body density. (Supplementary Figure S5B, right panel). PROseq also yields information on long eRNA transcription. THZ1 treatment yielded nine long eRNAs that had increased gb polymerase density along with concomitant increase in polymerase pause at the start site (Figure 5D, top panel). For the 34 long eRNA that showed decreased gb density, there was only a modest increase of polymerase pausing at the start site (Figure 5D, bottom panel). The overall distribution of PROseq signal of gb up and gb down long eRNA, are shown in Supplementary Figure S5C and D respectively.

Next, we ranked super-enhancers using the H3K27ac signal from ChIP-exo analysis (Figure 5E) (31,72–73). With THZ1 treatment, we observed a modest rise in the number of polymerases at THZ1-treated super-enhancers (Figure 5F). This was also true for *RUNX1*, as rather than a loss of RNA polymerases within the super-enhancer; there was an enhancement in polymerase density (Supplementary Figure S3A, *RUNX1* gene track). Similar changes were observed in the *MYC* super- enhancer where THZ1 stimulated the accumulation of polymerases within the body of the major eRNA (Supplementary Figure S5E). Gene body density for the genes associated with either typical (TE) or super-enhancers (SE) was assessed using a closest proximity rule (31,73). Genes associated with super-enhancers showed an increase in RNA polymerases in the gene body after THZ1 treatment when compared to typical enhancers (Figure 5G). An example of increased polymerase density in the gene body and the nearby enhancer is shown at *SPI1* locus (Figure 5H). Thus, the rapid decline of Kasumi-1 cells was not due to loss of polymerase in the body of the fusion gene or at the *RUNX1* super-enhancer.

### THZ1 treatment affects RNA polymerase dynamics at the 3′ end of the genes

**Figure 6.**
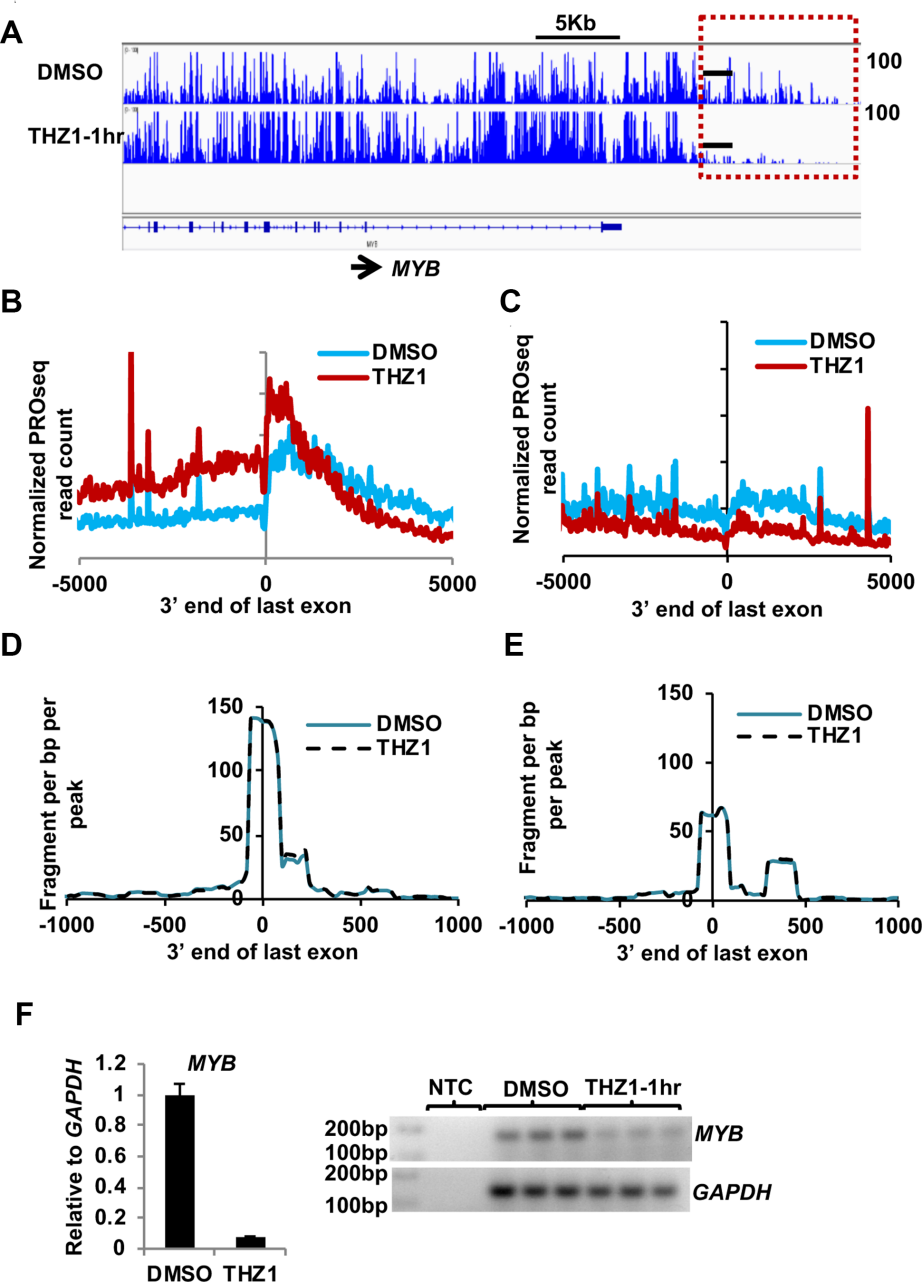
THZ1 affects transcription at the 3′ end from the last exon. (**A**) PROseq IGV browser screenshot of genome tracks for *MYB* after 1 h of THZ1 treatment. Red dashed box highlights the region 3′ to the last exon and black bars indicate the region where primers were designed for the qRT-PCR. (**B, C**) Metaplots of normalized PRO-seq read signal for THZ1 treated samples at 1 h was plotted ±5 kb of the last exon divided into 20 bp bins for the genes with increased gene body density from Figure 3B, 944 genes (third panel), or genes with decreased gene body density from Figure 3B, 1070 genes (third panel). (**D, E**) Metaplots of normalized PolyA-seq read signal for THZ1 treated samples at 1 h was plotted ±1 kb from the last exondivided into 20 bp bins for the above mentioned genes in (C). (**F**) Graph of qRT-PCR of the effected region (*n* = 3) from *MYB* shows decreased read through transcript levels after 1 h treatment with THZ1. The representative gel image shows the qRT-PCR endpoint for technical replicates from one qRT-PCR experiment.

The C-terminal domain of RNA polymerase II couples RNA transcription to splicing and polyadenylation processes to produce a fully functional mRNA (49,74). Ser2 phosphorylation of the CTD contributes to 3′ end processing (75,76), while phosphorylation of Ser5 of the CTD by CDK7 was associated with termination, cleavage and polyadenylation of mRNAs as well as with the recruitment of SSu72, a phosphatase that targets the CTD to recycle the polymerase (77). In our analysis of PROseq data, we noted that in the control cells, RNA polymerase runs on past the end of the last exon but in the THZ1 treated cells, we found that those genes with more gene body polymerase after THZ1 treatment showed a marked skewing of polymerase toward the end of the last exon (Figure 6A). In a meta-analysis of these genes (the 944 genes from Figure 3B, THZ1), we found that polymerase peaks centered near the end of last exon or just 3′ to this region and rapidly declined 3′ of this region (Figure 6B). However, in genes that were inhibited by THZ1, there was no obvious change (Figure 6C).

To test whether THZ1 influences the selection of the site of 3′ RNA cleavage and polyadenylation, we used poly A site anchored RNA-sequencing (PolyA-seq) a method used to map 3′ cleavage and poly A sites (36). We found that for most genes the first poly A site was the predominant site used, and this did not change in the presence of THZ1 (Figure 6D and E). This was true whether we first subdivided the genes based on increased polymerase density or loss of transcription after THZ1 treatment. (Figure 6D and E). Thus, it appears that inhibiting CDK7 may have slowed elongation of RNA polymerase near the end of the last exon in a subset of genes, rather than altered poly A site selection. These effects were confirmed using qRT-PCR of selected genes such as *MYB* (Figure 6F).

### THZ1 treatment affects RNA polymerase dynamics at the 5′ and 3′ ends of genes

CDK7 plays a critical role at the 5′ end of genes to pause RNA polymerases and enhance 5′ capping (Figure 3) (17), but it has also been suggested to travel down the gene body with RNA polymerase (78,79). Given our PROseq results that THZ1 affected polymerase dynamics at the ends of genes (Figure 6), we attempted to determine if CDK7 functions at the 3′ ends of genes. Because RNA polymerases move down genes at roughly 2–4 kb/min (31), we reanalyzed the time course of THZ1 treatment reasoning that if THZ1 affects CDK7 in the gene body or at the 3′ end of genes, these effects should be observed quickly (within 15 to 30 min) at the end of both short genes and genes greater than 200 kb in length.

**Figure 7.**
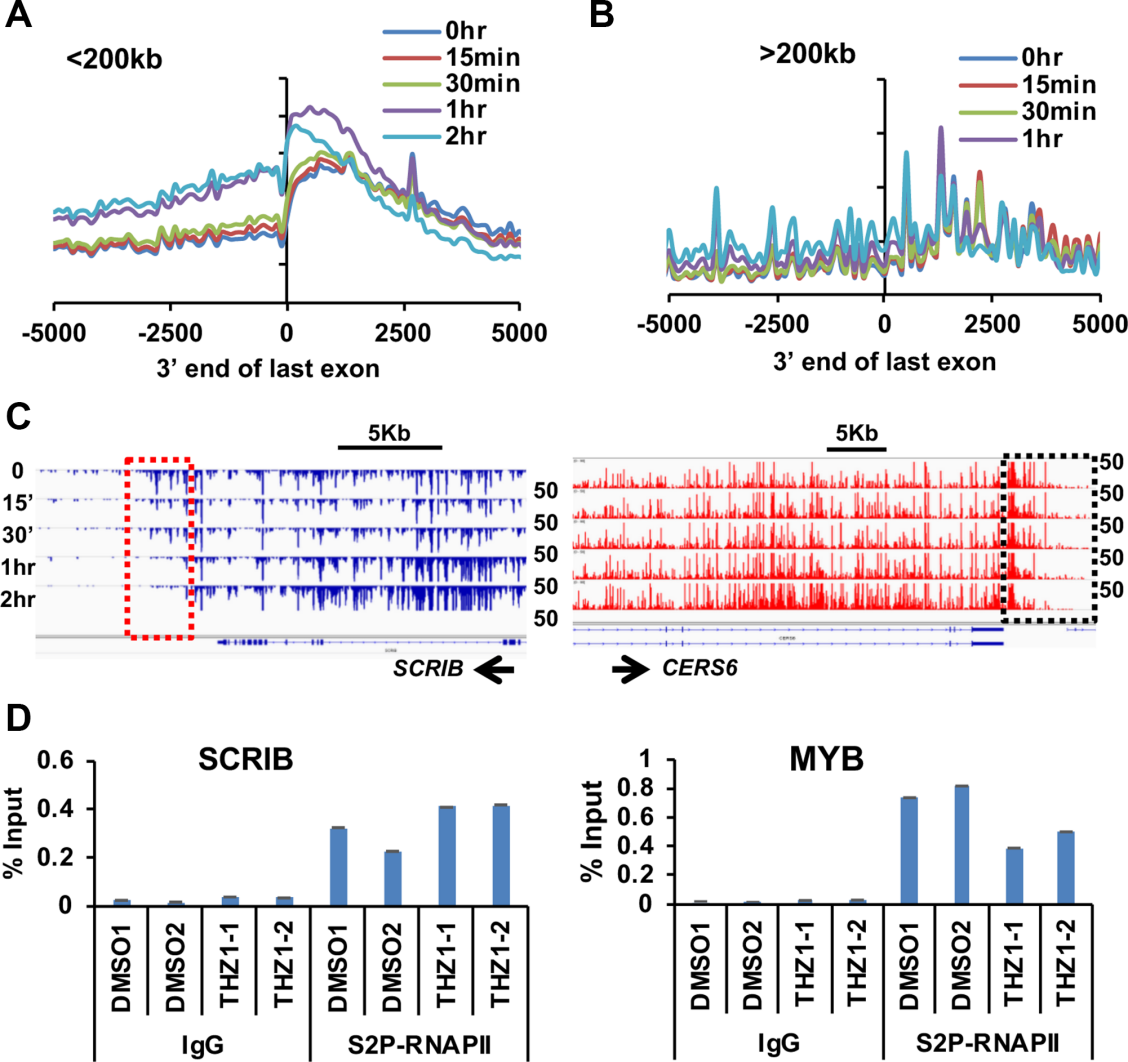
THZ1 affects both 5′ and 3′ end polymerase pausing. (**A**) Meta-analysis of short genes (less than 200 kb long) with more polymerase in the gene body at 1 h after THZ1 treatment plotted ± 5 kb from the 3′ end of the last exon at the times indicated after THZ1 treatment. (**B**) Same analysis as in (A) but using genes over 200 kb long. (**C**) IGV browser screenshots of genome tracks for a short gene *SCRIB* and a long gene *CERS6*. (**D**) Ser2-RNAPII ChIP-qPCR was performed after 1 h THZ1 treatment for *SCRIB* and *MYB*. The primers were designed at the regions just 3′ of the last exon. Two biological replicates of either DMSO or THZ1 treated Kasumi-1 cells were used. Error bars are mean ± SD of replicates. See Supplemental Figure S6 for additional examples.

For the analysis of polymerase dynamics at the 3′ end of genes, we divided the genes with higher polymerase gene body density at 1 h (Figure 3D) into genes shorter or longer than 200 kb. For short genes, some loss of polymerase ‘read through’ was noted within 15′ and this effect became more prominent at 30′ and 1 h (Figure 7A, *SCRIB* is an example in Figure 7C, see also PLXNA1 in Supplementary Figure S6A). However, for long genes where any polymerases that would be modified at the start site had not yet had time to reach the 3′ end at the early time points, polymerase dynamics around the termination site were not greatly affected in the first 30′ or even 1 h in some cases (Figure 7B, right panel; 7C, right panel shows a long gene, *CERS6* = 319 kb see also *MKL1* in Supplementary Figure S6B). The effects on ‘read through’ transcription most closely matched the timing of polymerases modified at the 5′ end of the gene eventually reaching the 3′ end of the genes. Moreover, there were no consistent effects on RNA polymerase C-terminal domain Ser2 phosphorylation at the ends of affected genes as assessed by chromatin immunoprecipitation (Figure 7D and Supplementary Figure S6C). These results suggest that inhibiting CDK7, possibly by impairing the phosphorylation of Ser5 at the 5′ end of the gene, eventually affected polymerase dynamics at the 3′ end of many genes.

## DISCUSSION

CDK inhibitors offer an attractive therapeutic approach to treating AML containing the t(8;21), as cells containing this translocation were rapidly killed by inhibitors of CDK9, as well as CDK7 (Figure 1). It is notable that these AML cells were much more sensitive to the broad-spectrum CDK inhibitors flavopiridol and dinacilib than to a more selective CDK9 inhibitor (PHA767491). In fact, at the levels where PHA767491 was active (5 uM), it likely inhibits cell cycle CDKs to some degree. AML containing t(8;21) are associated with stabilizing mutations of *CCND2* and inactivating mutations of *MGA* (44,46–47) which promote the G1 to S phase transition, but palbociclib had little effect, even in combination with PHA767491 (negative data not shown). These results imply that other cell cycle kinases are responsible for this t(8;21)-selective killing by flavopiridol and dinaciclib with the most likely candidates being CDK1 and CDK2.

By analyzing RNA polymerase dynamics after short treatments with inhibitors of kinases that regulate the cell cycle and/or transcription, we identified a mechanism for CDK7 action at most of the transcribed genes. Upon CDK7 inhibition we observed that about 80% of the genes transcribed displayed attenuated pausing at promoters (Figure 3). These results agree with chemical genetics analysis using a mutated CDK7, which is sensitive to by adenine analogs in HCT116 cells where a few selected genes were tested (9,10) and *in vitro* studies using reporter genes (10). However, the initial report of THZ1 suggested that it caused a genome-wide loss of RNA Pol II. By using ChIP-seq for RNA pol II that does not yield directional information and by using later time points, it is possible that the promoter proximal pause release was not observed (16). After longer durations of treatment with THZ1, secondary effects may come into play as we observed increases in annexin V positive populations within the first 2–4 h after THZ1 treatment (Figure 1G). This inhibition also impaired the phosphorylation of RNA Pol II CTD Ser2 within four hours of treatment, while CDK9 inhibition reduced the CTD Ser2 phosphorylation in as little as 1hr (Figure 2B). This delay is likely due to CDK7-dependent activation of CDK9 (CDK7 is the CAK for CDK9), as CDK7 is more effective in phosphorylating the CTD at Ser5 and Ser7 (80), while CDK9 prefers Ser2. This indirect inhibition of CDK9 likely underlies our observations that THZ1 also caused promoter-proximal pausing of some genes (Figure 3).

Although the loss of polymerase at transcriptional start sites and promoter proximal regions could be interpreted as an initiation block (28,51), a block in initiation would cause loss of gene body read densities and diminished RNAPII occupancy throughout the first 100 kb of genes within an hour. In contrast, our PROseq results indicated that only a few genes showed this effect with the majority of affected genes showing an increase of gene body polymerase read densities. The more modest level of transcript accumulation as depicted by RNA-seq could be due to the short duration of treatment used (1hr) that would not allow a large accumulation of cytoplasmic pools of poly-adenylated mRNA. This interpretation is consistent with prior work using an in vitro system in which THZ1 treatment compromised co-transcriptional capping and loading of DSIF and NELF on to the pre-initiation complexes (17). However, it is also possible that inhibition of CDK7 slowed the elongation rate of polymerase, causing it to accumulate in the gene body. Nevertheless, our data are consistent with CDK7, along with NELF and DSIF, mediating pause establishment on the majority of genes and when it is inactivated polymerase leaks into the gene body while initiation continues.

The chemical genetics analysis of mutated CDK7 also suggested that CDK7 inhibition not only affected promoter-proximal polymerase pausing, but also delayed termination and poly A site selection. However, this analysis used only chromatin immunoprecipitation of RNA Polymerase II using primer pairs along 3 different genes (9,10). In contrast, our whole genome PROseq analysis indicated that the opposite is true on a significant number of genes where THZ1 reduced the amounts of active polymerases downstream of thelast exon. A major advantage of PROseq is that it yields directional information whereas ChIP-seq does not. This is critical where genes are in close proximity and this might have affected results for genes such as *GAPDH* (9,10) where a second gene is juxtaposed. Intriguingly, a similar phenotype was observed with a mutant RNA polymerase II with decreased elongation efficiency (29,30). In fact, a reduced elongation rate after THZ1 treatment would also explain the narrowing of the ‘gap’ around the transcription start site over time after THZ1 treatment (Figure 3), as polymerases ‘pile up’ within the first 5 kb of the start site. This time course analysis suggests that the effects of inhibiting CDK7 occur at the 5′ ends of genes, yet the decrease in elongation rate does not appear to affect completion of transcription as these polymerases appear to reach the 3′ ends of genes (Figure 3).

While it has been recognized that t(8;21) AML is very sensitive to flavopirodol for over 20 years (81), our study suggests that CDK7 inhibitors will also benefit these patients. By dissecting the transcriptional mechanisms of these two classes of compounds genome-wide, it appears that these drugs work in opposing manners. At the transcriptional level, CDK9 inhibitors cause promoter proximal RNA polymerase pausing, which causes highly transcribed genes that are also tightly controlled by mRNA and protein turnover (such as *MYC*) to be rapidly lost (82). In contrast, CDK7 inhibition caused the loss of pausing at the 5′ promoter-proximal region, but this was associated with ‘enhanced pausing’ at the 3′ end of genes. The major conundrum is that CDK7 acts as the cyclin activating kinase for multiple cell cycle CDKs as well as CDK9 (83), so over time its inhibition has pleiotropic effects and might mimic flavopiridol, but with slightly delayed kinetics. If this latter point holds true, the clinical development of flavopirodol might portend the clinical development of THZ1 and similar compounds.

## DATA AVAILABILITY

The accession number for PROseq and RNA-seq data is GEO: GSE102243. NRSA pipeline for PROseq analysis: http://bioinfo.vanderbilt.edu/NRSA/.

## Supplementary Material

Supplementary Data
