# Supplementary material for: The CDK7 inhibitor THZ1 alters RNA polymerase dynamics at the 5′ and 3′ ends of genes
Source: Nucleic Acids Res. 2019 Feb 26;47(8):3921–36. doi: 10.1093/nar/gkz127 (PMC6486546; doi:10.1093/nar/gkz127)
Supplement: Supplementary Data [file gkz127_supplemental_files.zip › Top25_1hrgbupMotifs.pdf]

Rank	Name	Motif	% with Motif	P-value
1	Sp1	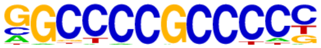	46.14%	1.00E-267
2	Elk4(ETS)	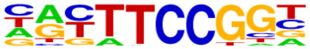	35.59%	1.00E-209
3	NFY(CCAAT)	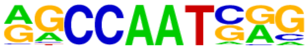	25.92%	1.00E-200
4	Elk1(ETS)	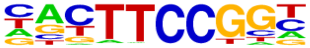	34.87%	1.00E-197
5	ETS(ETS)	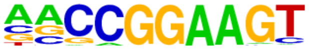	23.70%	1.00E-190
6	ELF1(ETS)	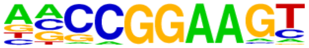	32.38%	1.00E-188
7	Fli1(ETS)	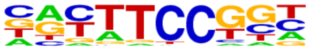	40.43%	1.00E-177
8	GABPA(ETS)	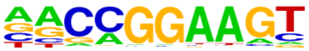	32.93%	1.00E-173
9	ETV1(ETS)	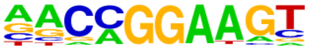	38.83%	1.00E-148
10	KLF5(Zf)	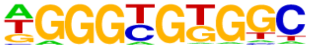	63.33%	1.00E-147
11	Klf9(Zf)	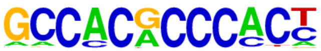	31.53%	1.00E-138
12	ETS1(ETS)	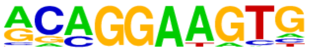	31.18%	1.00E-132

Rank	Name	Motif	% with Motif	P-value
13	KLF14(Zf)	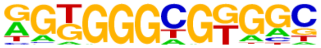	73.43%	1.00E-110
14	NRF(NRF)	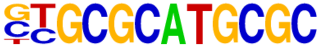	18.52%	1.00E-105
15	Etv2(ETS)	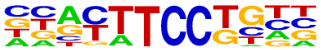	24.30%	1.00E-100
16	EHF(ETS)	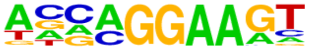	26.94%	1.00E-96
17	ERG(ETS)	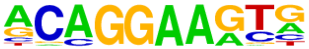	34.00%	1.00E-95
18	NRF1(NRF)	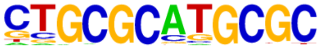	20.34%	1.00E-92
19	GFY-Staf	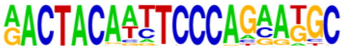	6.83%	1.00E-90
20	EWS:FLI1-fusion(ETS)	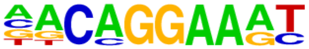	17.57%	1.00E-85
21	YY1(Zf)	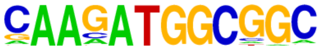	8.15%	1.00E-81
22	ELF5(ETS)	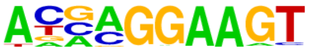	17.32%	1.00E-75
23	Klf4(Zf)	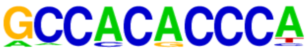	19.17%	1.00E-71
24	GFY	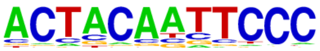	5.78%	1.00E-64
25	CRE(bZIP)	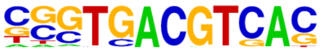	11.07%	1.00E-60
