# Supplementary figures and images for: The CDK7 inhibitor THZ1 alters RNA polymerase dynamics at the 5′ and 3′ ends of genes
Source: Nucleic Acids Res. 2019 Feb 26;47(8):3921–36. doi: 10.1093/nar/gkz127 (PMC6486546; doi:10.1093/nar/gkz127)

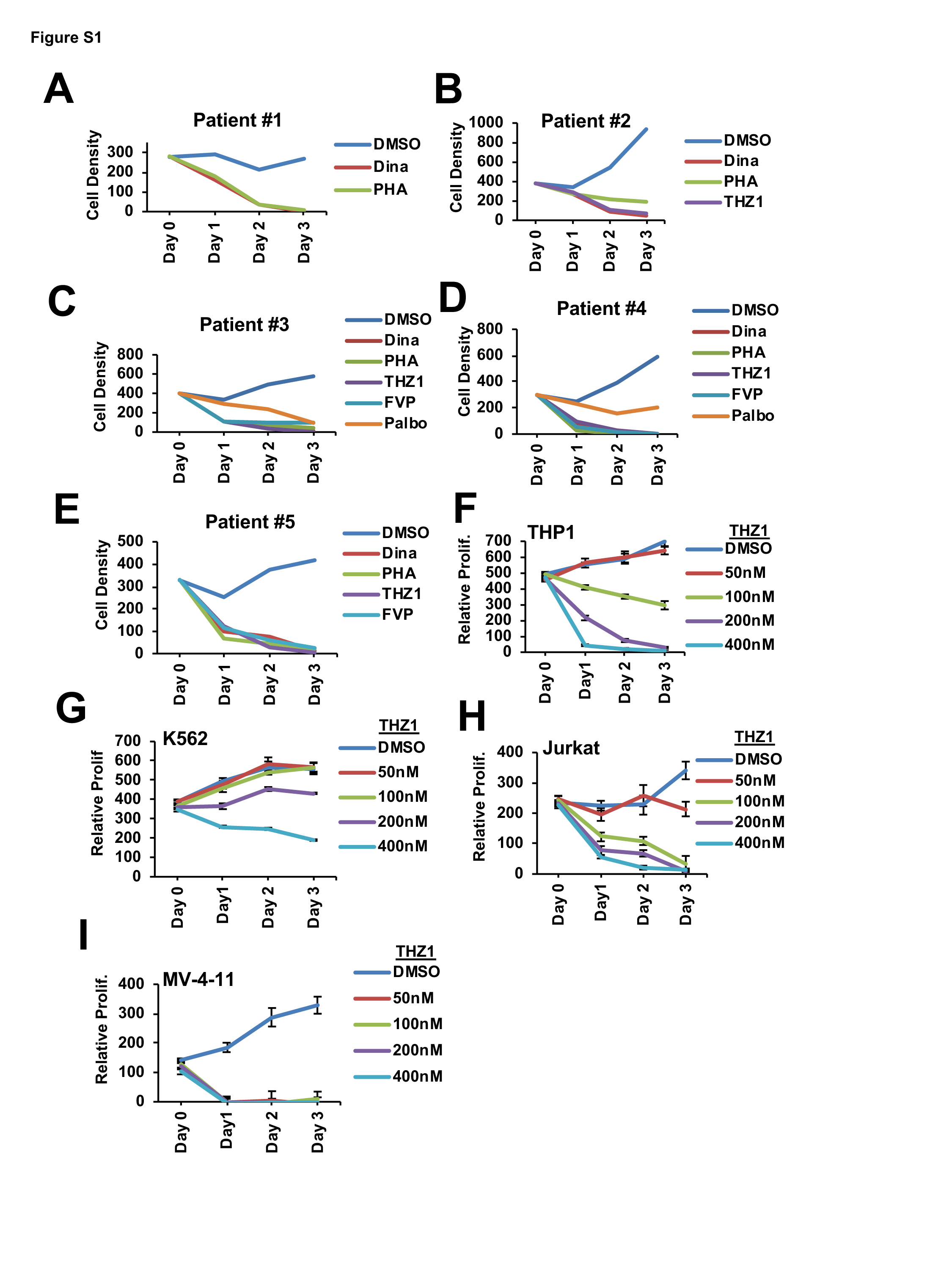

Supplement: Supplementary Data [file gkz127_supplemental_files.zip › THZ1_supplemental figures_NAR_revised-1.tif]

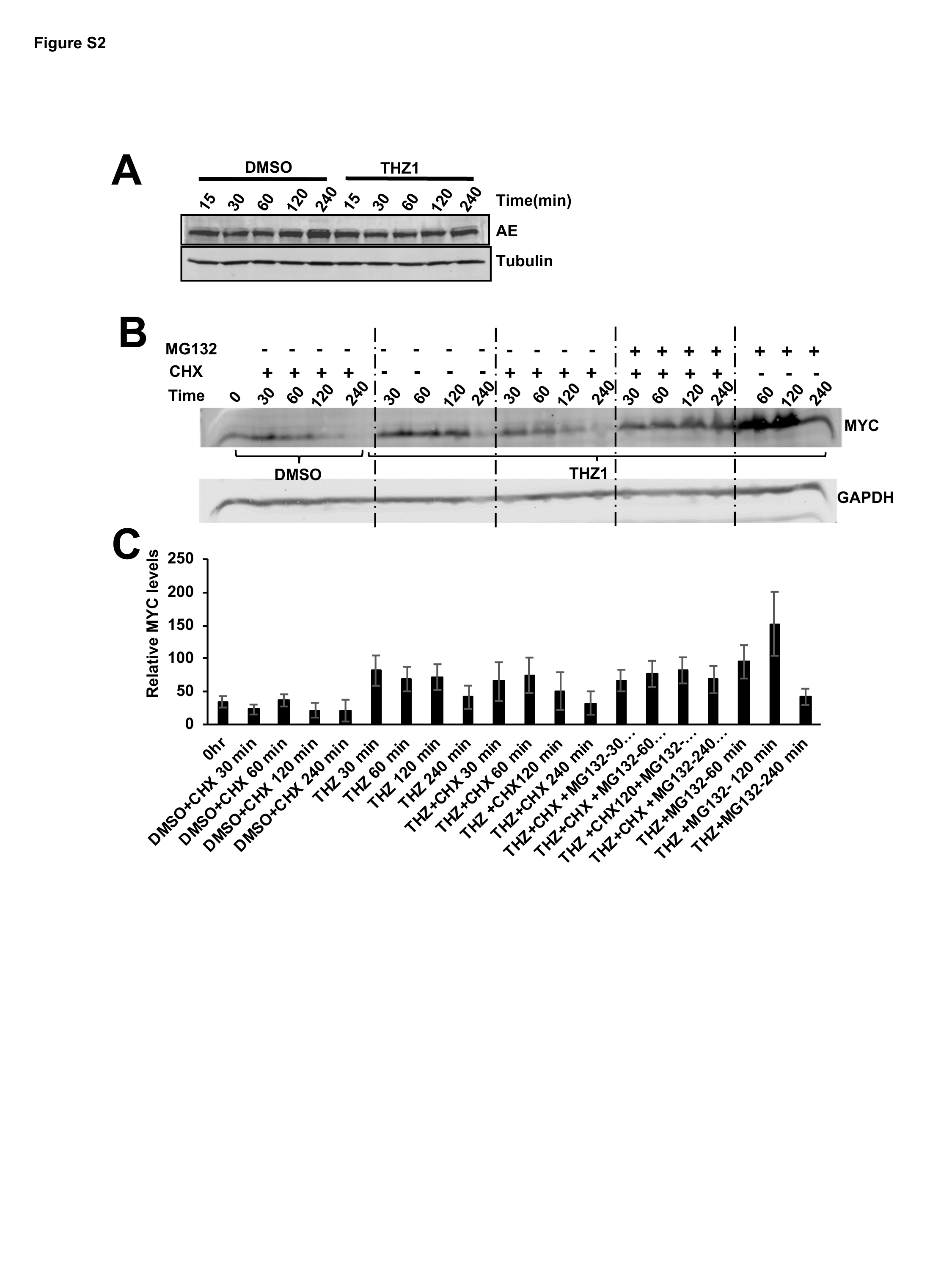

Supplement: Supplementary Data [file gkz127_supplemental_files.zip › THZ1_supplemental figures_NAR_revised-2.tif]

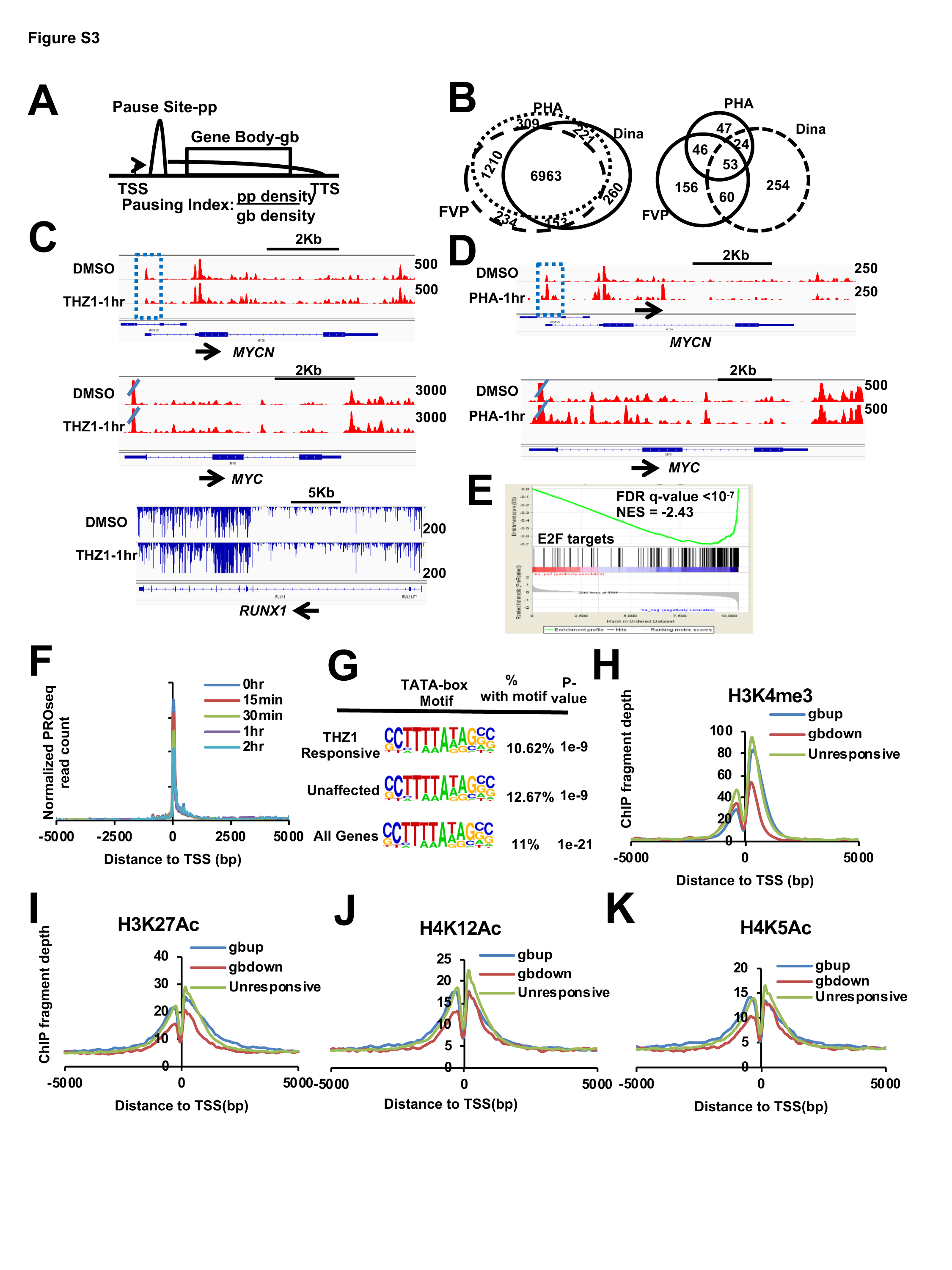

Supplement: Supplementary Data [file gkz127_supplemental_files.zip › THZ1_supplemental figures_NAR_revised-3.tif]

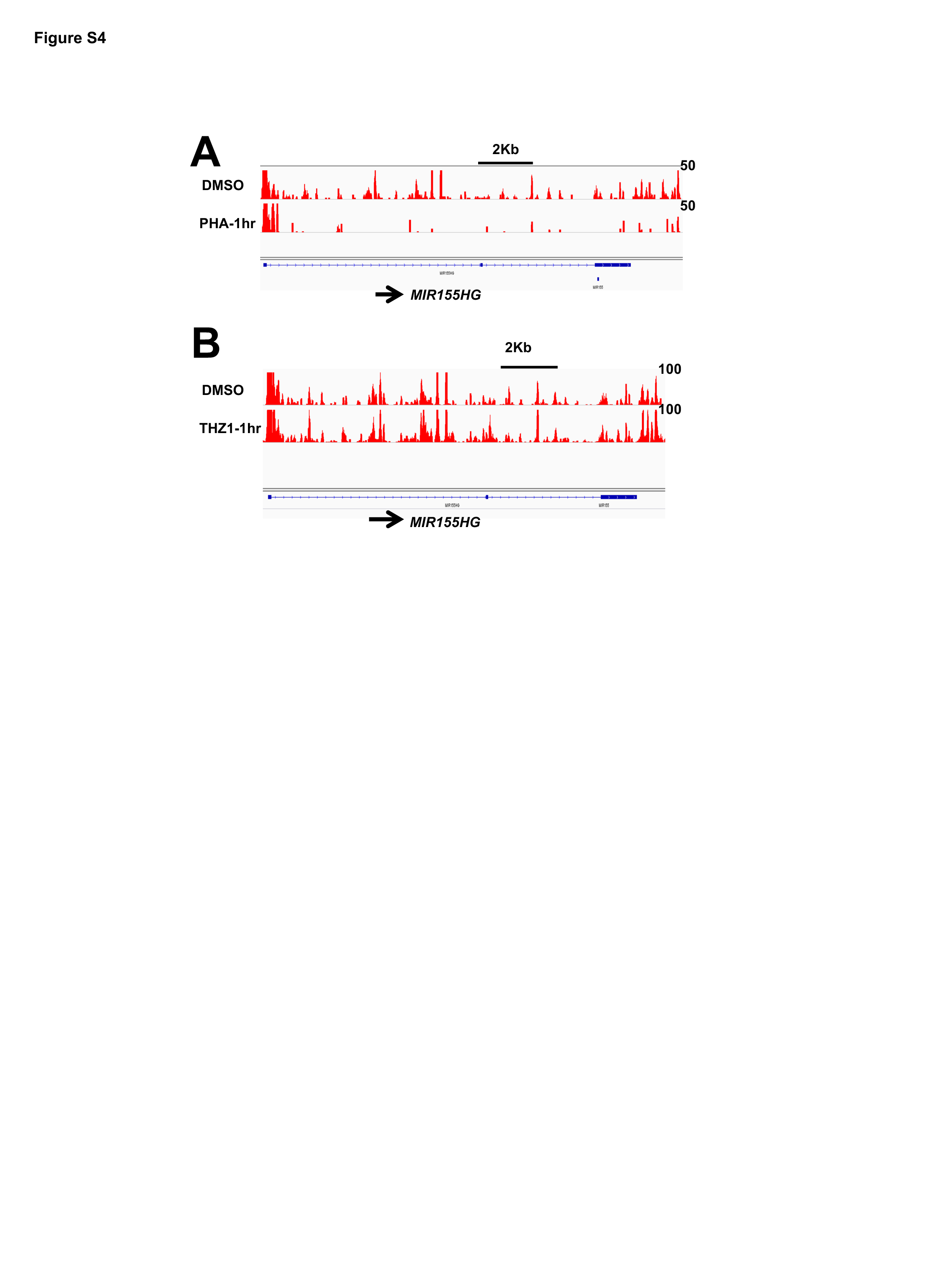

Supplement: Supplementary Data [file gkz127_supplemental_files.zip › THZ1_supplemental figures_NAR_revised-4.tif]

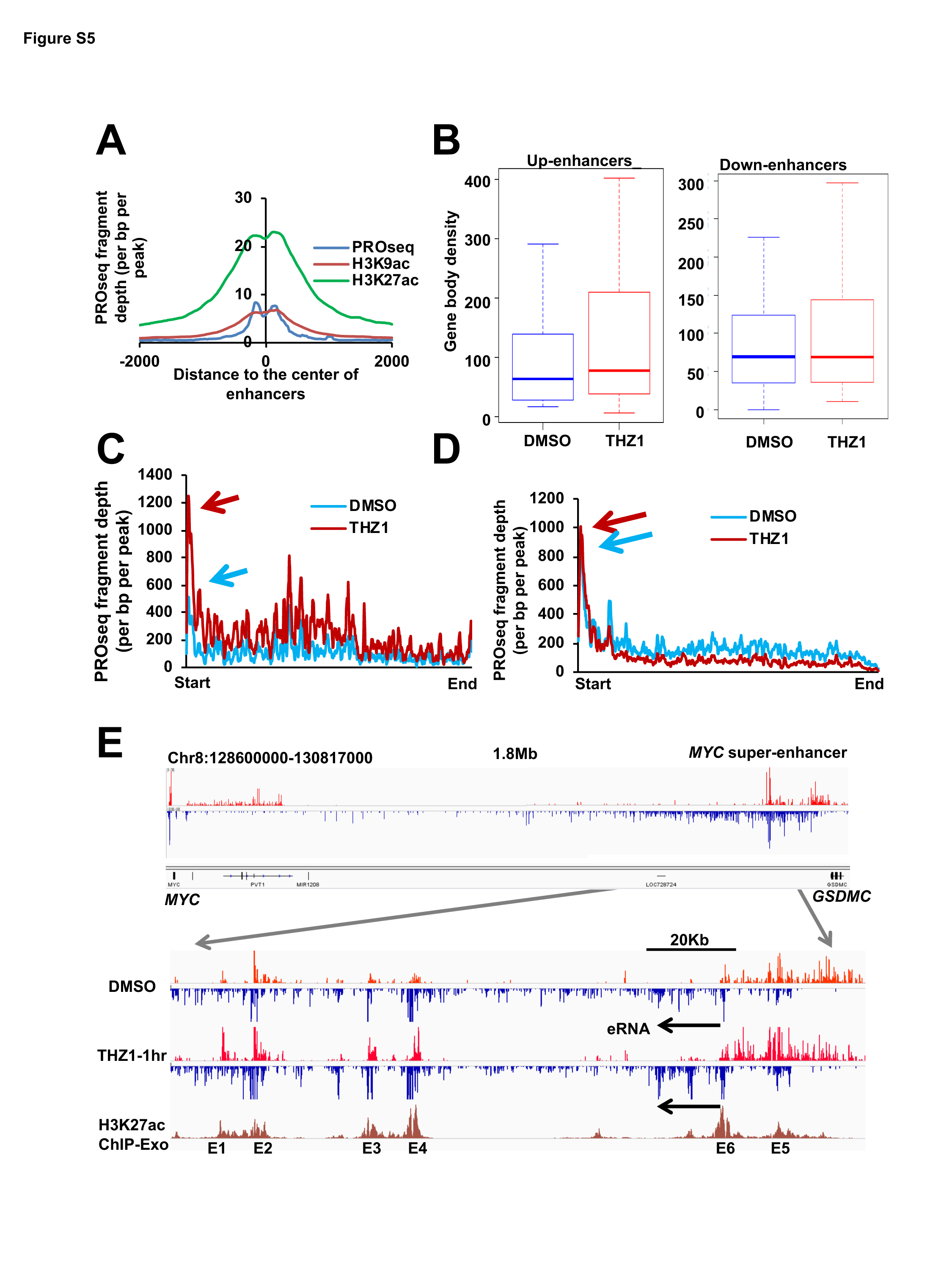

Supplement: Supplementary Data [file gkz127_supplemental_files.zip › THZ1_supplemental figures_NAR_revised-5.tif]

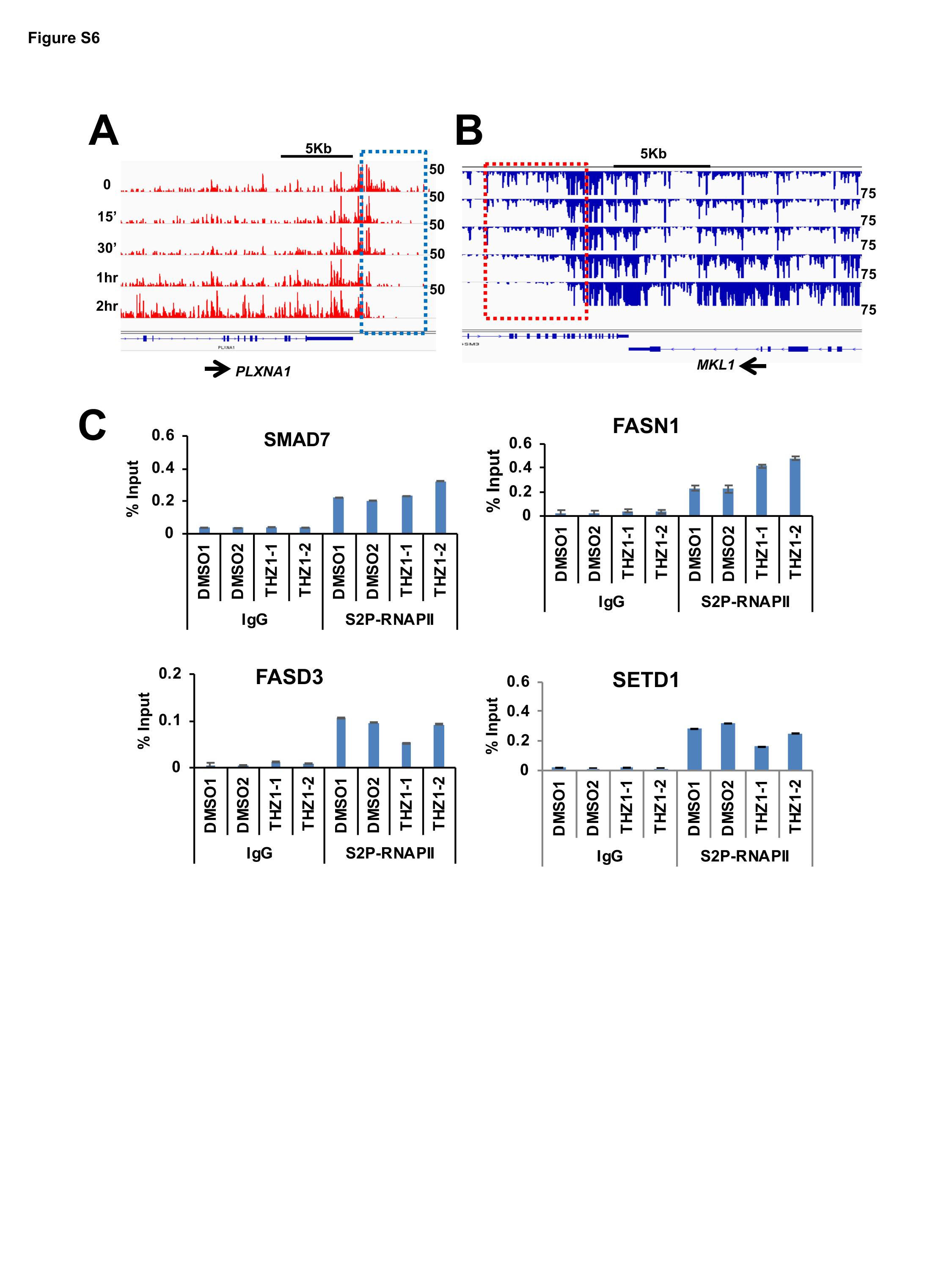

Supplement: Supplementary Data [file gkz127_supplemental_files.zip › THZ1_supplemental figures_NAR_revised-6.tif]

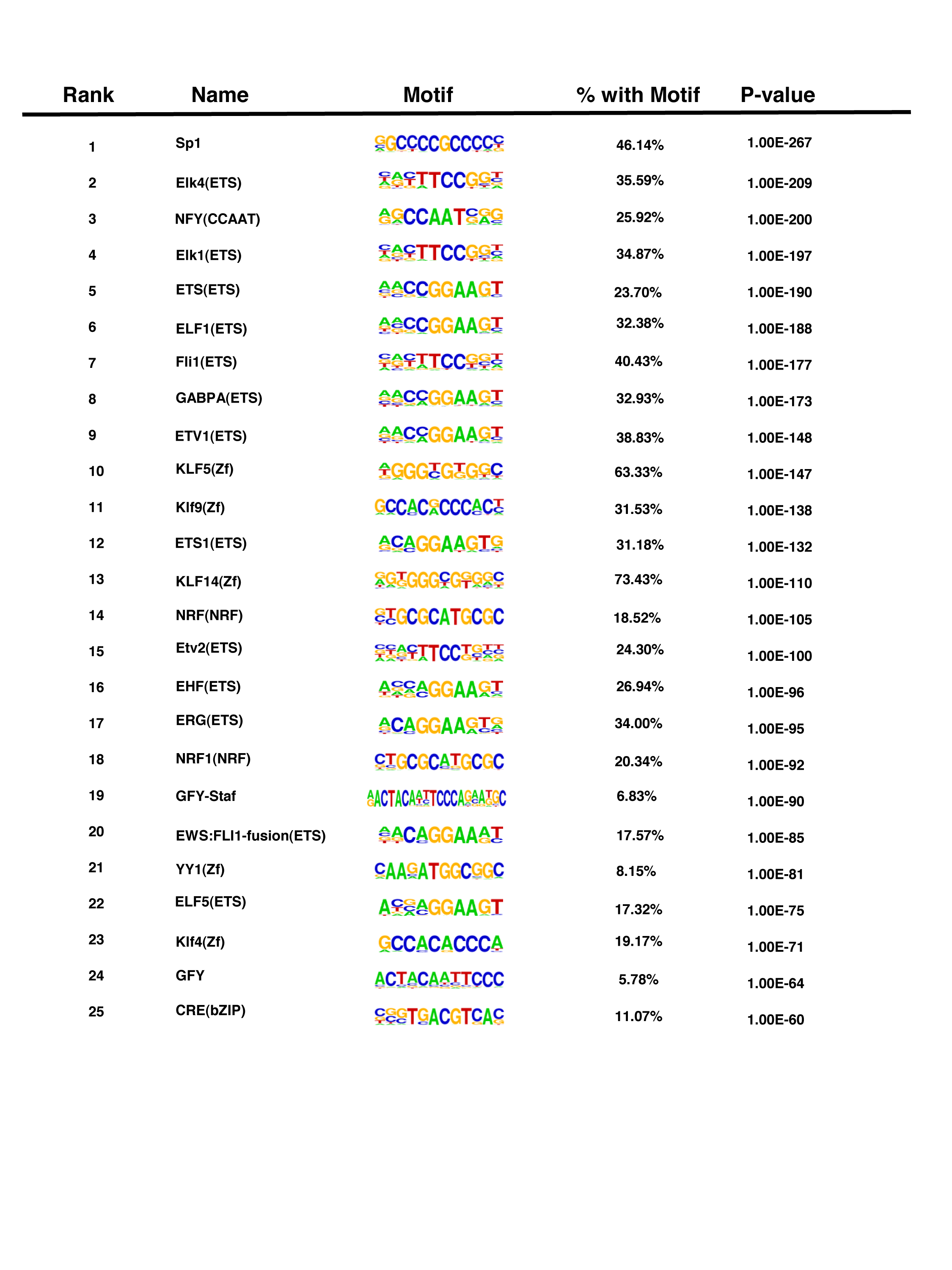

Supplement: Supplementary Data [file gkz127_supplemental_files.zip › Top25_1hrgbupMotifs.tif]
